# Imagination in Medicine

**Published:** 1896-01

**Authors:** Stuart Close


### IMAGINATION IN MEDICINE.

EDITOR OF THE HOMŒOPATHIC PHYSICIAN :

Since the publication of my article on " Imagination in Medicine," in the October issue of THE HOMŒOPATHIC PHYSICIAN, I have received letters which indicate that I was in error in not clearly defining the term *imagination* at the outset.

Taking it for granted that the meaning of the term was generally understood, I proceeded to point out the evils arising from its misuse, and to plead for the proper and scientific use of this faculty in Homœopathic Medicine.

It is sufficient to say that the article has been misunderstood and the importance of the subject greatly underrated.

Its importance can only be appreciated by a clear understanding of the true nature of the faculty under discussion. I propose, therefore, to supplement that article, by your courtesy, with some definitions and quotations drawn from standard authorities, that it may be seen that I have not only used the term in the accepted sense, but that the position taken is consistent with such usage and with the needs of the case.

Let it be premised, then, that the imagination is a normal and necessary faculty of the mind ; that like any other faculty it may be misused and lead to evil, but that its right use must redound to the highest good of its possessor. What is this faculty and what its sphere of action ?

"Imagination: the act or faculty of forming a mental image of an object; the act or power of presenting to consciousness objects other than those directly and at that time produced by the action of the senses. The act or power of reproducing or recombining remembered images of sense objects, especially the higher form of the power exercised in poetry and art. Commonly divided into reproductive and productive; reproductive being the power or faculty of reproducing images stored in the memory under the suggestion of associated images; productive being the creative imagination, which designedly recombines former experiences into new images.

"In the Kantian philosophy, used to denote the pure transcendental imagination, or that faculty by which the parts of the intuitions of time and space are combined into continua."—*Century Dictionary.*

In all of these uses it is to be observed that the use of this faculty always depends upon experience and knowledge, even in what is termed reproductive or creative imagination, where its so-called creative power is simply exercised in recombining previous experiences or ideas into new forms. It originates no conceptions. Its materials are the results of previous perceptions and conceptions stored up in memory.

Discrimination must be made between Imagination and Fancy. They were formerly used synonymously. They are frequently confused, even now, though they have long since come to have clearly-defined lines of separation. "Imagination is profound, earnest, logical. Fancy is lighter, more sportive, more often purely creative."

"It is evident that true imagination is vastly different from fancy; far from being merely a playful outcome of mental activity, a thing of joy and beauty only, *it performs the initial functions in every branch of human development.*" (Italics mine.) —*Maudsley, Body and Will, page* 201.

My own idea is that the comparison between Imagination and Fancy is superfluous, owing to a misconception of the true nature of the faculty under discussion. They are spoken of as two faculties. There is but one faculty—imagination. That faculty may be exercised either in the realm of facts or the realm of fancy. In the one case it is a serious and responsible use, in the other a light and sportive use of the one primary faculty, the difference being merely in its field of action.

"The faculty of the mind by which it either bodies forth the forms of things unknown, or produces original thoughts, or new combinations of ideas, from materials stored up in the memory." — *Worcester's Dictionary.*

Dugald Stewart, the great Scottish philosopher, says:

"The faculty of imagination is the great spring of human activity and the principal source of human improvement." Also: "The business of conception is to present us with an exact transcript of what we have felt or perceived. But we have, moreover, a power of modifying our conceptions by combining the parts of different ones together so as to form new wholes of our own creation. I shall employ the word 'imagination' to express this power."

It will be evident, upon a little reflection, I think, that the last quotation describes the use made of the faculty by the homœopathician when he rightly studies a proving or makes a prescription. Symptoms of the sick are kaleidoscopic, never arranging themselves twice alike. As they are constantly forming new combinations, so must the prescriber form corresponding new combinations from the materials in the provings of drugs, in order, by comparison, to find the similar remedy. If he form these combinations in an arbitrary or mechanical manner he is likely to fail. They must be so combined as to form a consistent and harmonious whole. He can only form and recognize such a whole by the use of his Imagination. Such a conception is usually called the "genius" of the remedy or of the case.

The very act of comparison necessarily involves the use of the Imagination. No comparison can otherwise be made. The symptoms of the patient cannot be compared with the symptoms of the proving until both symptom records are thus pictured before the mind for its action and judgment, not only in detail, but as a whole.

Tyndall, in his celebrated address before the British Association at Liverpool, on "The Scientific Use of the Imagination," began by quoting the eloquent words of Sir Benjamin Brodie, as follow:

"Lastly, physical investigation, more than anything else, helps to teach us the actual value and right use of the Imagination—of that wondrous faculty which, left to ramble uncontrolled, leads us astray into a wilderness of perplexities and errors, a land of mists and shadows; but which, properly controlled by experience and reflection, becomes the noblest attribute of man; the source of poetic genius, the instrument of discovery in science, without the aid of which Newton would never have invented fluxions, nor Davy have decomposed the earths and alkalies, nor would Columbus have found another continent."

Tyndall himself says:

"With accurate experiment and observation to work upon, imagination becomes the architect of physical theory. Newton's passage from a falling apple to a falling moon was an act of the prepared imagination, without which the Laws of Kepler could never have been traced to their foundation. * * * Without the use of this power our knowledge of nature would be a mere tabulation of co-existences and sequences. We should still believe in the succession of day and night, of summer and winter; *but the conception of force would vanish from the universe; causal relations would disappear, and with them that science which is now binding the parts of nature into an organic whole.*"

Without the exercise of this sublime faculty, HAHNEMANN would never have formed his conceptions of a vital force, of high potencies, or of a Psora theory.

These definitions and quotations appear sufficient to emphasize the importance of the subject I attempted to treat, and to justify the endeavor to make the application of this faculty to the science of Homœopathy more definite and more general. It has been shown that imagination must be used to enable us to use the knowledge we have obtained, by observation and experience, to the best advantage, in homœopathic philosophy, as well as in all other branches of science and art. How it is to be used in Homœopathy I tried to show in my paper.

What more need be said, therefore, in reply to one who declared to me in a letter that "with knowledge, imagination is not required. Imagination can never guide to truth. With the discovery of Homœopathy there is no further use for imagination in medicine, but only facts!"

STUART CLOSE, M. D.

